# A Simple Method to Identify Kinases That Regulate Embryonic Stem Cell Pluripotency by High-throughput Inhibitor Screening

**DOI:** 10.3791/55515

**Published:** 2017-05-12

**Authors:** Charles A. C. Williams, Nathanael S. Gray, Greg M. Findlay

**Affiliations:** ^1^The MRC Protein Phosphorylation and Ubiquitylation Unit, School of Life Sciences, University of Dundee; ^2^Department of Cancer Biology, Dana-Farber Cancer Institute; ^3^Department of Biological Chemistry and Molecular Pharmacology, Harvard Medical School

**Keywords:** Developmental Biology, Issue 123, Protein Kinase, Embryonic Stem Cell, Pluripotency, Naïve-Primed Transition, Kinase Inhibitors, High-Throughput Screen

## Abstract

Embryonic stem cells (ESCs) can self-renew or differentiate into all cell types, a phenomenon known as pluripotency. Distinct pluripotent states have been described, termed "naïve" and "primed" pluripotency. The mechanisms that control naïve-primed transition are poorly understood. In particular, we remain poorly informed about protein kinases that specify naïve and primed pluripotent states, despite increasing availability of high-quality tool compounds to probe kinase function. Here, we describe a scalable platform to perform targeted small molecule screens for kinase regulators of the naïve-primed pluripotent transition in mouse ESCs. This approach utilizes simple cell culture conditions and standard reagents, materials and equipment to uncover and validate kinase inhibitors with hitherto unappreciated effects on pluripotency. We discuss potential applications for this technology, including screening of other small molecule collections such as increasingly sophisticated kinase inhibitors and emerging libraries of epigenetic tool compounds.

**Figure:**
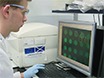


## Introduction

Embryonic stem cells (ESCs) have the capacity to self-renew or differentiate into any cell type in the adult body, a phenomenon known as pluripotency^1^. Recent evidence indicates that developmentally distinct pluripotent states exist, termed "naïve" and "primed" pluripotency^2^. Naïve ESCs represent a state of development similar to that found in the preimplantation embryo^3^. In contrast, primed pluripotent ESCs are poised to exit pluripotency and differentiate into specialized embryonic lineages^4^,^5^.

Naïve and primed pluripotent states are marked by distinct gene regulatory networks. Naïve pluripotency is characterized by expression of key pluripotency transcription factors such as Nanog, Krueppel-like transcription factors (Klfs), Rex1^2^ and Esrrb^6^. In mouse ESCs (mESCs), primed pluripotency is characterized by reduced expression of naïve markers and a specific gene expression signature which includes the de novo DNA methyltransferase Dnmt3b^7^. *In vivo*, primed pluripotent post implantation epiblast stem cells (EpiSCs)^4^,^5^ additionally express the Epiblast marker Fgf5 and markers of lineage priming such as Brachyury^8^.

mESCs provide a tractable model to probe mechanisms that control naïve-primed pluripotent transitions *in vitro*. When cultured in leukemia inhibitory factor (LIF) and fetal bovine serum (FBS), mESCs undergo dynamic transition between naïve and primed pluripotent states^9^,^10^. LIF-Jak-Stat3 signaling functions to promote a naïve gene regulatory network^11^, whilst autocrine signaling via the fibroblast growth factor 4 (Fgf4) Erk1/2 pathway drives transition to the primed state^12^. However, it remains a challenge to systematically evaluate the role of protein kinases in specifying distinct pluripotent states.

Here, we describe a quantitative and scalable platform by which to perform targeted small molecule screens for kinase regulators of the naïve-primed pluripotent transition. We use simple mESC culture conditions and standard reagents, materials and equipment to uncover and validate kinase inhibitors with hitherto unappreciated ability to stabilize naïve pluripotency. Furthermore, we discuss potential extended applications for this technology, including for screening of other small molecule collections such as emerging inhibitor libraries targeting epigenetic regulators.

## Protocol

### 1. Collation of Small Molecule Kinase Inhibitor Libraries

1. Collate inhibitor libraries from publicly accessible kinase inhibitor collections, *e.g.* Library of Integrated Network-based Cellular Signatures (LINCS) from Harvard Medical School, a targeted collection of 228 potent and selective kinase inhibitors (http://lincs.hms.harvard.edu), and the Protein Kinase Inhibitor Set (PKIS), a 376 compound collection assembled by industrial researchers (https://www.ebi.ac.uk/chembldb/extra/PKIS)^13^. Alternatively, assemble a bespoke kinase inhibitor collection from commercially available compounds. NOTE: In our laboratory, we have assembled a collection of 72 potent and selective tool kinase inhibitors covering 51 primary target kinases, all of which are available from commercial sources.
2. Assemble kinase inhibitors into 96-well plates for optimal high-throughput processing at multiple concentrations (use 1, 0.1 and 0.01 mM). Include in each plate control wells containing DMSO only and reference control inhibitors PD173074 (FGFRi) and Ruxolitinib (JAKi), which are known to promote naïve and primed pluripotent states respectively. Annotate in a spreadsheet or similar software.

### 2. mESC Culture Conditions and Procedures for Screen Preparation

1. Prepare 0.1% gelatin-coated 10 cm dishes by adding 5 mL of 0.1% (w/v) gelatin to 10 cm plates and incubate at room temperature for 5 min. Aspirate gelatin and let plate dry for 2 min.
2. Culture any standard mESC line at 37 °C 5% CO_2_ on 0.1% gelatin-coated 10 cm dishes in standard mESC media (see **Materials Table**) containing 100 ng/mL GST-LIF, 10% Fetal Calf Serum and 5% Knockout Serum Replacement. Replace media every day and passage mESCs at around 80% confluency every second day at a dilution of 1:5-1:10.
3. To passage mESCs, aspirate media and wash with 5 mL of phosphate-buffered saline (PBS) per plate.
4. Add 1 mL trypsin-EDTA (0.05% Trypsin, 0.02% EDTA) per plate of mESCs and incubate at 37 °C for 10 min.
5. Resuspend trypsinized cells in 4 mL of mESC media and centrifuge at 1,200 rpm for 5 min.
6. Thoroughly resuspend cell pellet in 5 mL of mESC media, pipetting up and down to generate a single cell suspension.
7. Count cells by combining a 10 µL cell suspension and 10 µL of Trypan Blue (0.4%). Place into a cell counting chamber or use a hemocytometer and light microscope.
8. Seed 3 x 10^3^ mESCs into 0.1% gelatin coated 96 well plates, final volume 100 µL of media, using a multichannel pipette.
9. Apply kinase inhibitors at 1:100 dilution (1 µL inhibitor added to 100 µL media) using a multi-channel pipette. Gently pipette media to mix inhibitor and cell suspension, then allow cells to settle in a tissue culture hood for 1 h to ensure equal distribution across the plating surface. Culture cells for 48 h without changing the media. NOTE: Stock plates of 1/0.1/0.01 mM will give a final inhibitor concentration of 10/1/0.1 µM respectively. we recommend starting the screen at a final concentration of 1 µM to ensure effective inhibition of primary target kinases whilst minimizing off-target effects.

### 3. Kinase Inhibitor Screening Analysis

1. Wash 96 well mESC plates in 200 µL PBS using multi-channel aspirator and pipettes.
2. Make cell extracts in 150 µL of lysis buffer (20 mM Tris pH 7.4, 150 mM NaCl, 1 mM EDTA, 1% NP-40 (v/v), 0.5% sodium deoxycholate (w/v), 10 mM β-glycerophosphate, 10 mM sodium pyrophosphate, 1 mM NaF, 2 mM Na_3_VO_4_, Complete Protease Inhibitor Cocktail Tablets).
3. Clarify extracts by centrifugation at 1,500 x g for 30 min in V-bottomed 96-well plates.
4. Immobilize 100 µL of supernatants onto a nitrocellulose membrane using a 96-well vacuum dot blot manifold.
5. Dry the membrane, and stain with 40 mL of Ponceau S to ensure consistent transfer.
6. Wash membrane with TBST and block in TBST/3% (w/v) milk.
7. Incubate in Nanog and Dnmt3b antibodies at a dilution of 1:1,000 (v/v) in TBST/3% (w/v) milk powder overnight.
8. Wash membranes in 3 x 10 min in TBST and incubate in 30 mL of secondary antibodies at a dilution of 1:10,000 (v/v) in TBST/3% (w/v) milk.
9. Develop using a digital immunoblotting imaging system. NOTE: If possible, use quantitative fluorescence immunoblotting to detect Nanog and Dnmt3b. However, the background signal for Dnmt3b by fluorescence immunoblotting is too high. Therefore, 800 nm fluorescence anti-rabbit (Nanog) and anti-mouse-HRP (Dnmt3b) secondary antibodies are used.

### 4. Analysis of Screen Data and Validation of Inhibitors as Bona Fide Pluripotency Regulators

NOTE: This is a critical step to ensure that only bona fide pluripotency modulators are identified. It is essential to triage inhibitors based on both Nanog:Dnmt3b ratio and overall signal, to ensure that kinase inhibitors which adversely affect mESC survival are not selected for further analysis.

1. Quantify Nanog and Dnmt3b signal using immunoblotting analysis software, and import values into a spreadsheet or similar software. Calculate Nanog:Dnmt3b ratio for all samples compared to DMSO control. Also, determine total Nanog and Dnmt3b signal to ensure that kinase inhibitors do not affect Nanog:Dnmt3b ratio by altering mESC viability.
2. Use ranked Nanog:Dnmt3b ratios to identify inhibitors which promote naïve pluripotency (Nanog:Dnmt3b ratio higher than control) and primed pluripotency (Nanog:Dnmt3b ratio lower than control). Set cut-off at 2x deviation from control values, and filter out kinase inhibitors which produce a total Nanog+Dnmt3b signal of less that 50% of the DMSO control value to generate a high confidence list of kinase inhibitors which stabilise naïve (high Nanog:Dnmt3b ratio) and primed (low Nanog:Dnmt3b ratio) pluripotent states.
3. Validate kinase inhibitors by seeding mESCs at 2 x 10^5^ cells per 6 well in 2 mL media, and add kinase inhibitors at 1 µM for 48 h, without changing media.
4. Lyse mESCs in lysis buffer and analyze Nanog and Dnmt3b expression by conventional SDS-PAGE immunoblotting.
5. Further validate hits by SDS-PAGE immunoblotting for naïve pluripotency markers Klf2 and Klf4, and Oct4 to ensure that pluripotency is maintained following 48 h inhibitor treatment.

## Representative Results

Using the procedure presented here (**Figure 1**), we screen the LINCS library of 228 potent and selective kinase inhibitors to identify those which modulate mESC pluripotency. The library is prepared at a concentration of 0.1 mM for a 1:100 dilution and final screening concentration of 1 µM in mESCs. 48 h later, mESCs were lysed and extracts prepared for quantitative dot blot analysis of Nanog and Dnmt3b expression (**Figure 2**, top). Results from other screening concentrations are not represented here for brevity. Nanog:Dnmt3b ratio is determined for each kinase inhibitor and compounds ranked and a 2x cut-off applied (**Figure 2**, bottom). Total Nanog and Dnmt3b signal relative to control is overlaid onto inhibitor ranking and subjected to a 0.5x cut-off, to allow triaging of inhibitors which show significant mESC toxicity. Select kinase inhibitors which stabilize the naïve state are validated using conventional Nanog/Dnmt3b immunoblotting (**Figure 3**). The primary kinase targets of these inhibitors are presented in **Table 1**. We also demonstrate the applicability of this screen to identify inhibitors which stabilize the primed state^14^.


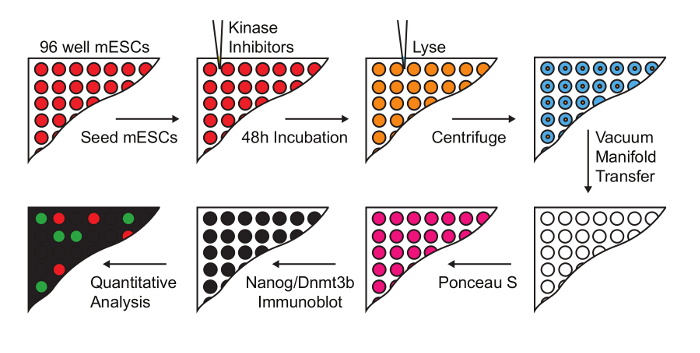
**Figure 1: Workflow for screening kinase inhibitors that modulate naïve-primed transition. **mESC were treated with a 228 compound kinase inhibitor library at a final concentration of 1 µM. Lysates were prepared and transferred onto nitrocellulose membranes for immuno dot-blot analysis, and Nanog and Dnmt3b levels determined (Figure adapted from Williams *et al.*^14^). Please click here to view a larger version of this figure.


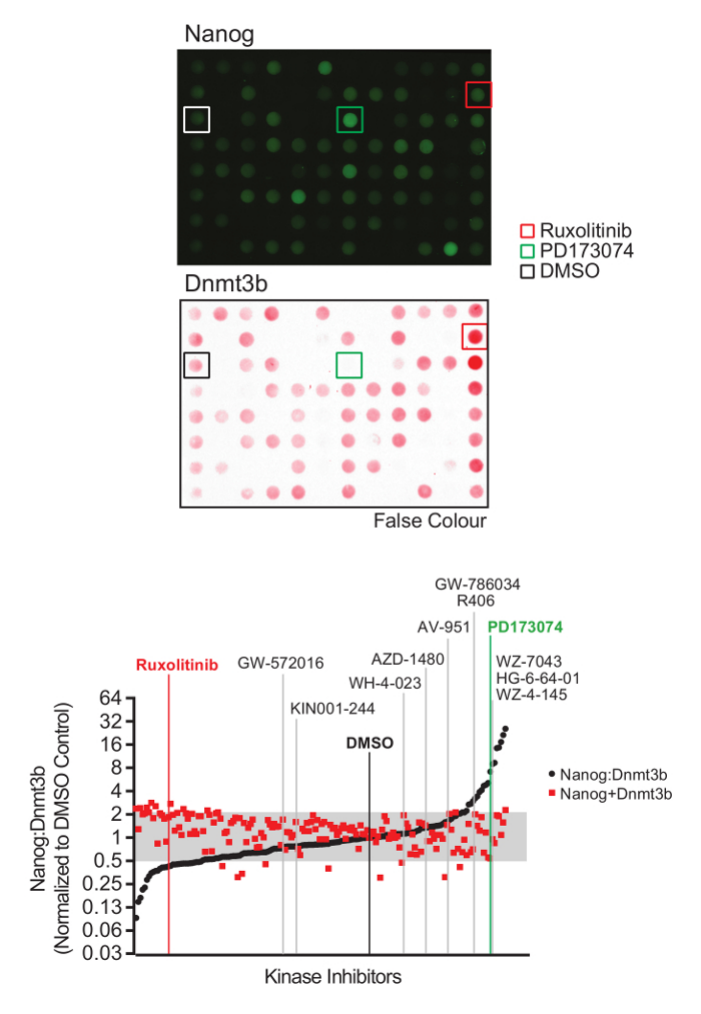
**Figure 2: Naïve-primed pluripotency screen analysis and inhibitor identification. **(Top) Representative Nanog and Dnmt3b immuno dot-blot images. (Bottom) Nanog and Dnmt3b values for each kinase inhibitor were determined relative to the DMSO control, and Nanog:Dnmt3b ratios and total relative Nanog+Dnmt3b signal determined and inhibitors ranked in comparison to DMSO control. Inhibitors found to alter Nanog:Dnmt3b ratio beyond a 2-fold threshold above or below DMSO control were identified as drivers of naïve or primed pluripotency. A threshold of 2x below DMSO control was set to triage inhibitors which compromise mESC survival. Inhibitors selected for further validation are highlighted. (Figure adapted from Williams *et al.*^14^). Please click here to view a larger version of this figure.


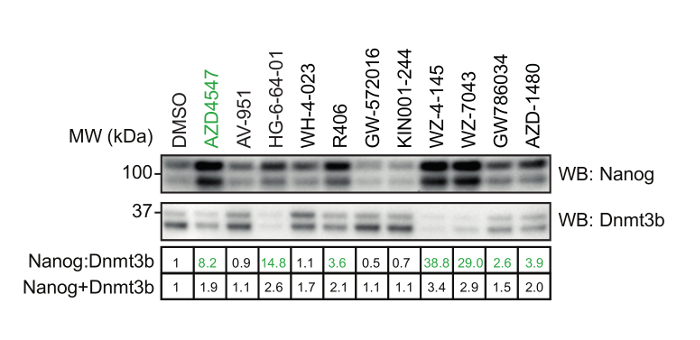
**Figure 3: Validation of selected hit compounds identified. **mESCs were treated with either 1 µM AZD4547 (a selective FGFR inhibitor) or the indicated inhibitors identified from the screen in Figure 2. Nanog:Dnmt3b levels determined by standard SDS-PAGE and immunoblot analysis. Numerical values of relative Nanog:Dnmt3b and Nanog+Dnmt3b are indicated in the table below, and inhibitors which stabilize the naïve pluripotent state highlighted in green. Please click here to view a larger version of this figure.

**Table:** 

**Compound**	**Primary Targets**	**Other Targets**
AV-951	VEGFR	
HG-6-64-01	ABL, BRAF, RET, CSF1R, EGFR, EPHA8, FGFR, FLT3, KIT, LOK, MAP4K1, p38b, MUSK,PDGFR, TAOK3, TNNI3K	
WH-4-023	Lck	Src
R406	Syk	
GW-572016	HER2	EGFR
KIN001-244	PDK1	
WZ-4-145	CSF1R, DDR1, EGFR, TIE1, PDGFR2	
WZ-7043	CSF1R, DDR1, FGFR and TAO1	
GW786034	VEGFR1	VEGFR2, VEGFR3
AZD-1480	JAK2	

**Table 1: Selected hit compounds and their primary kinase target(s).**

## Discussion

Here we demonstrate a widely accessible methodology to probe the role of kinase signalling pathways in regulating naïve-primed pluripotent transition. This addresses a key question in the ESC field. Although high-throughput genomics and transcriptomics approaches are routinely used to identify key transcriptional regulators of naïve and primed pluripotent states, elucidating pluripotency signalling networks has proven to be challenging. We now provide a flexible strategy employing chemical inhibitors to identify kinases that control naïve-primed pluripotent transition in mESCs. This relies on simple equipment, reagents and materials that are available to most laboratories that study cell signalling and ESC biology. Critical to the success of this platform is our development of a robust assay to quantify the transition between naïve and primed pluripotent states. Establishing this assay required significant iterative modifications to cell plating density, time of inhibitor incubation and immunoblotting conditions.

Small molecule approaches are gaining traction for rational use in therapeutic ESC applications^15^,^16^. A major strength of small molecule screening is that tool compounds are designed and/or modified for efficient cellular uptake. Transfection efficiency is not limiting, and use of hazardous delivery systems such as lentivirus is not necessary. Furthermore, inhibitors frequently inhibit multiple isoforms within a kinase family (*e.g.* Fgfr1-4, Jak1-3), which overcomes functional redundancy that hinders genetic/genomic interference techniques such as RNAi and CRISPR/Cas9. As more potent and selective tool compounds become available from both academic and pharmaceutical drug discovery programmes, understudied and poorly understood kinases will be pushed to the forefront of ESC research. We therefore propose that this high-throughput screening approach will open up new avenues of research into core ESC regulatory networks.

One minor limitation of this technology is that even the most potent and selective small molecule inhibitors engage and inhibit multiple unrelated kinases *in vivo*. However, increasingly comprehensive kinase inhibitor profiling data allows functional targets of kinase inhibitors to be readily identified (http://lincs.hms.harvard.edu/kinomescan; http://www.kinase-screen.mrc.ac.uk/kinase-inhibitors). Finally, in principle our platform can be applied to interrogate any small molecule collection and/or cellular assay for which high-quality immunoblotting antibodies are available. This will allow study of multiple regulatory systems in pluripotency regulation and facilitate identification of small molecule inhibitors which modify diverse cellular processes. Specifically, we envisage that application of emerging small molecule collections such as the epigenetic probes being developed by the Structural Genomics Consortium (http://www.thesgc.org/chemical-probes/epigenetics) has the potential to uncover further novel regulators of pluripotent transitions.

## Disclosures

The authors declare no conflicts of interest arising from this study.
